# Supplementary material for: A bacterial extracellular vesicle‐based intranasal vaccine against SARS‐CoV‐2 protects against disease and elicits neutralizing antibodies to wild‐type and Delta variants
Source: J Extracell Vesicles. 2022 Mar 14;11(3):e12192. doi: 10.1002/jev2.12192 (PMC8920961; doi:10.1002/jev2.12192)
Supplement: Supplementary file 1 — Supporting Information [file JEV2-11-e12192-s001.docx]

**Supplementary Information**

**Supplemental information 1**

Amino acid sequences of RBD constructs. Signal peptide in yellow, His-tag in magenta, SpyTag in light blue, and RBD in dark blue.

**His-Spy-RBD:**

**MFVFLVLLPLVSSQGSSHHHHHHGSGESGAHIVMVDAYKPTKGSGGTGRVQPTESIVRFPNITNLCPFGEVFNATRFASVYAWNRKRISNCVADYSVLYNSASFSTFKCYGVSPTKLNDLCFTNVYADSFVIRGDEVRQIAPGQTGKIADYNYKLPDDFTGCVIAWNSNNLDSKVGGNYNYLYRLFRKSNLKPFERDISTEIYQAGSTPCNGVEGFNCYFPLQSYGFQPTNGVGYQPYRVVVLSFELLHAPATVCGPKKSTNLVKNKCVNF****

**RBD-Spy-His**

**MFVFLVLLPLVSSQRVQPTESIVRFPNITNLCPFGEVFNATRFASVYAWNRKRISNCVADYSVLYNSASFSTFKCYGVSPTKLNDLCFTNVYADSFVIRGDEVRQIAPGQTGKIADYNYKLPDDFTGCVIAWNSNNLDSKVGGNYNYLYRLFRKSNLKPFERDISTEIYQAGSTPCNGVEGFNCYFPLQSYGFQPTNGVGYQPYRVVVLSFELLHAPATVCGPKKSTNLVKNKCVNFGSGGTGAHIVMVDAYKPTKGSGESGHHHHHH****


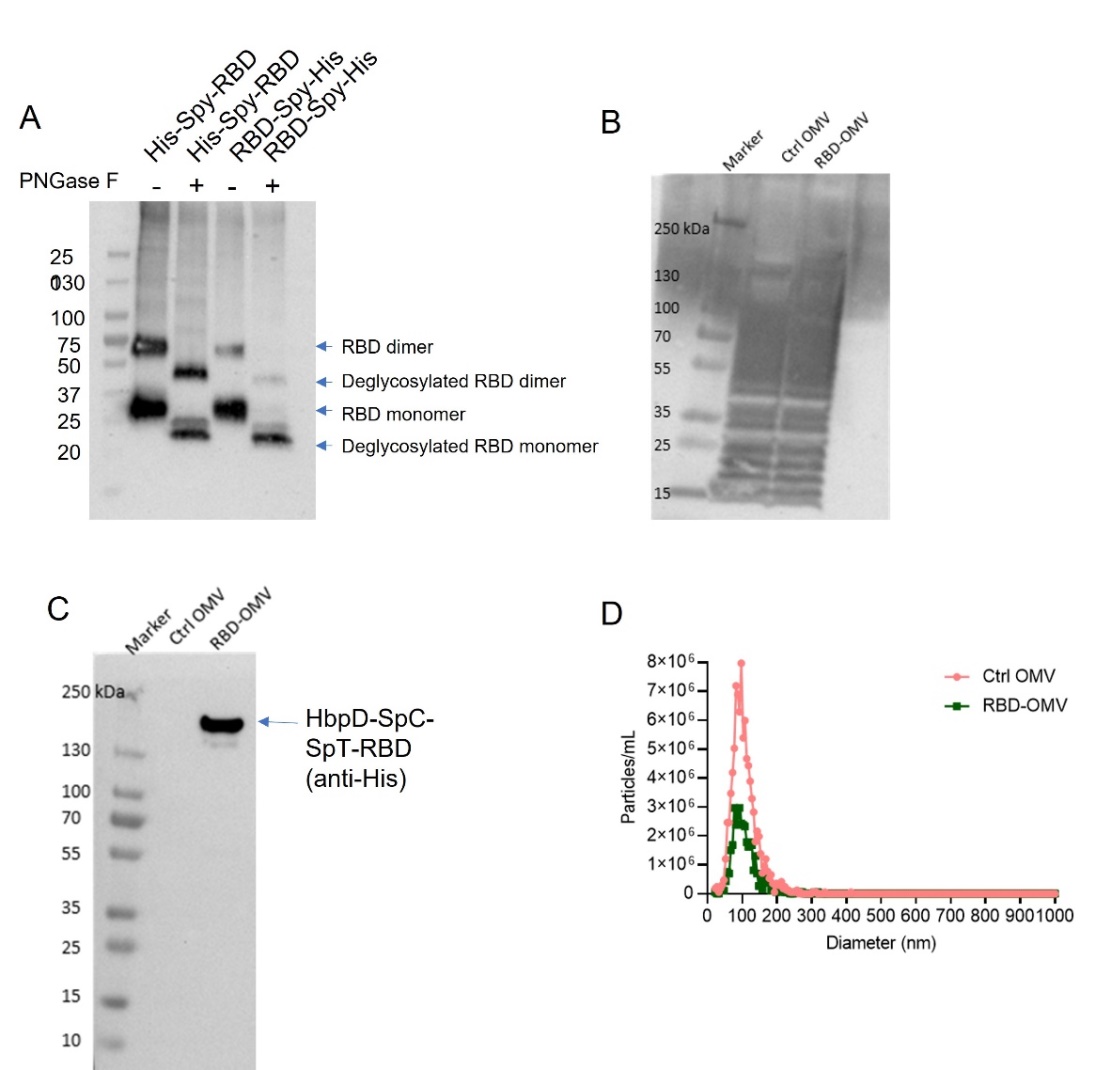


**Figure S1**. Additional bulk characterization of OMVs. A) Immunoblot results for RBD protein with/without PNGase F treatment; B) Western blot characterization of Ctrl-OMV and RBD-OMV with anti-LPS antibody. C) quantification of RBD in RBD-OMV by anti-His Western blot. D) Characterization of Ctrl-OMV and RBD-OMV by nanoparticle tracking analysis


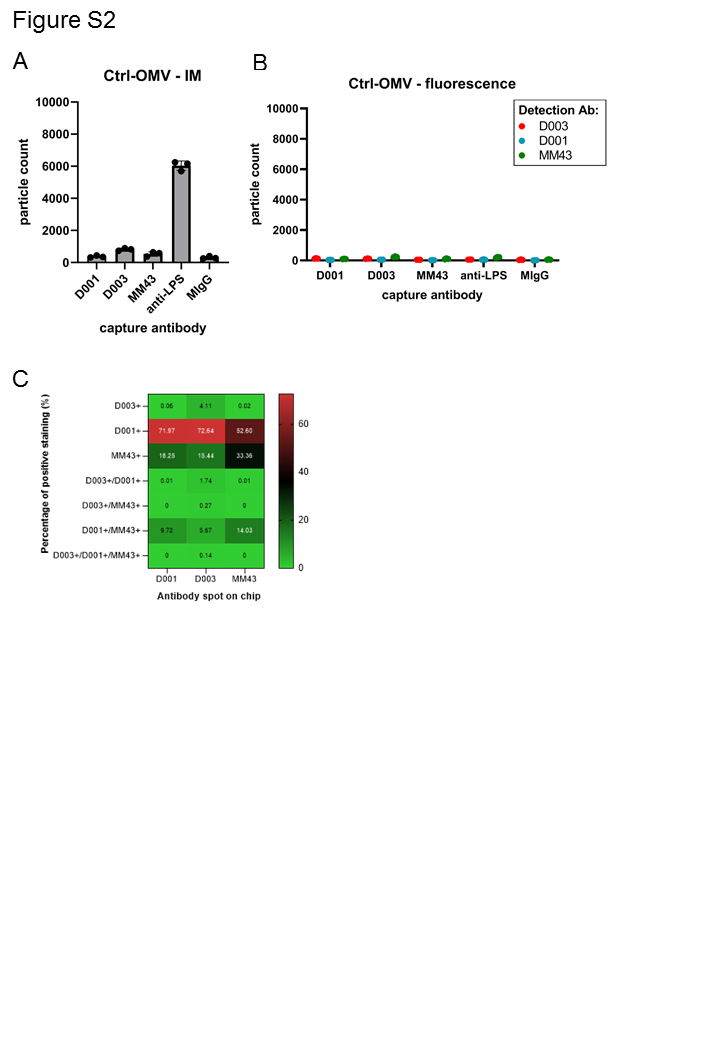


**Figure S2**. SP-IRIS results for Ctrl-OMVs. (A) Interferometric mode, (B) fluorescence mode. Datapoints show particle counts per capture spot, n=3 capture spots. (C) SP-IRIS results for RBD-OMV, corresponding to Figure 3D-E. Heatmap depicts the percentage of co-localization between fluorescent anti-Spike antibodies on RBD-OMV captured on SP-IRIS chips.


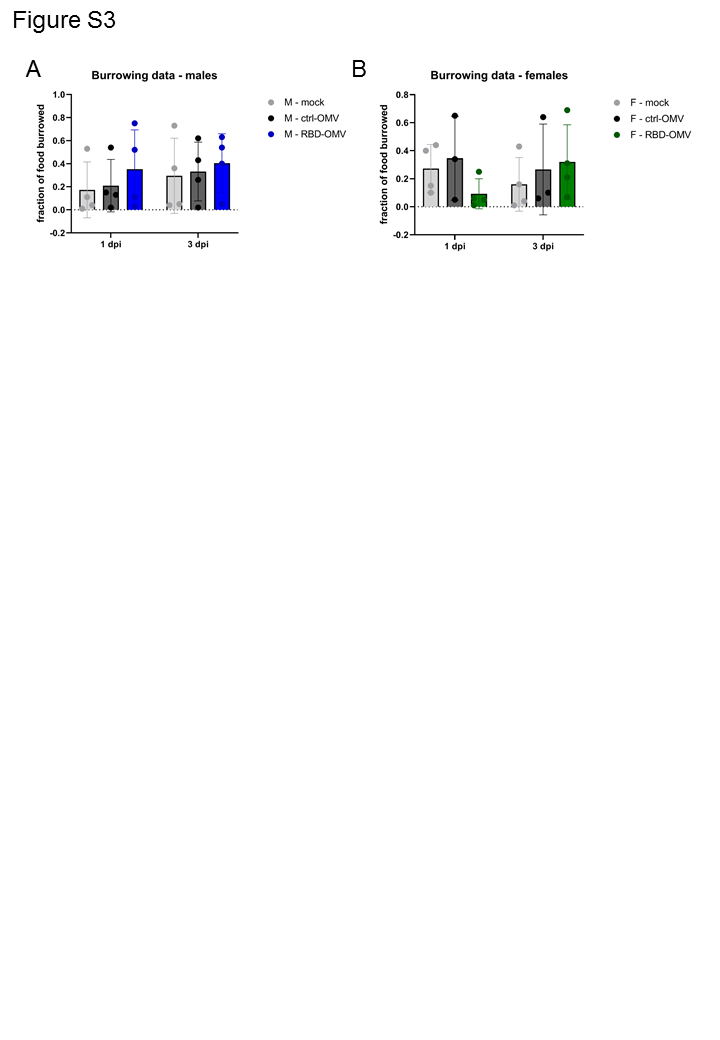


**Figure S3**. Food burrowing behavior was measured one and three days post-challenge. The fraction of burrowed food was determined by dividing the weight of food after overnight burrowing by the amount of food given to the animals. A) Males and B) females did not show statistical differences in burrowing behavior as analyzed by one-way ANOVA, n=4.


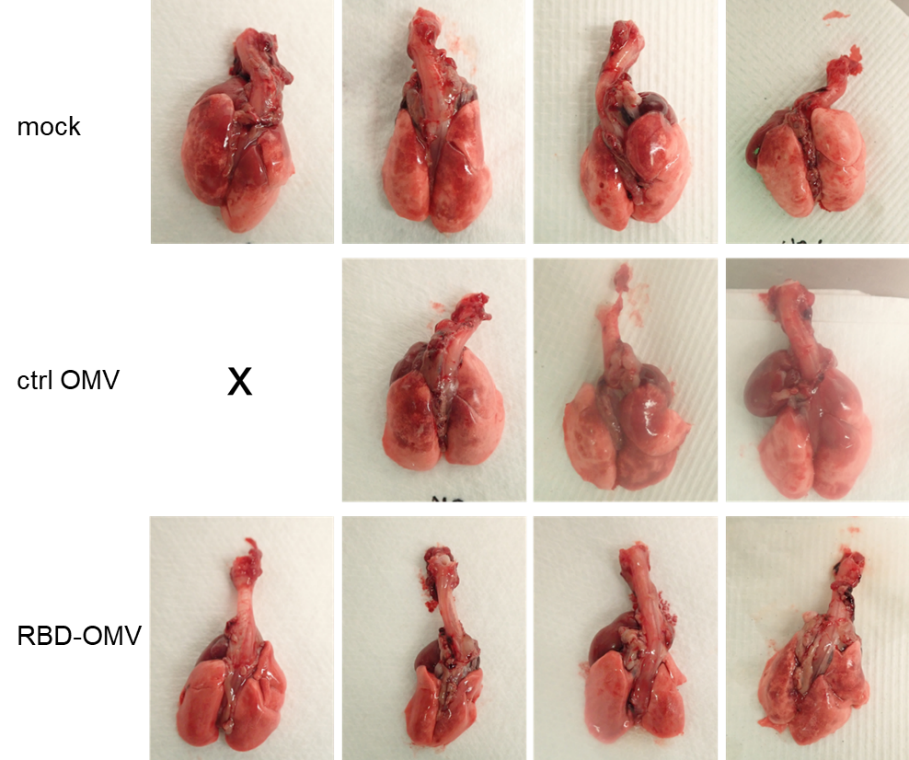


**Figure S4.** Lungs from female hamsters immunized with different formulations.
